# Case report: Duplicate renal ureteral malformation with renal tumor, ureteral tumor, renal rupture, calculi and infection

**DOI:** 10.1016/j.ijscr.2025.111907

**Published:** 2025-09-03

**Authors:** Maokun Sun, Baoqun Xu, Heng Zhang, Zhipeng Yan, Lihua Li, Yunji Sun

**Affiliations:** a Department of Urology, Shandong Provincial Third Hospital, Shandong University, Jinan, 250012, China; b Shandong Stone Disease Prevention and Treatment Center, Jinan, 250012, China; c Department of Pathology, Shandong Provincial Third Hospital, Shandong University, Jinan, 250012, China

**Keywords:** Duplex collecting system, Renal tumor, Ureteral tumor, Kidney stone, Urothelium carcinoma, Synchronous carcinomas

## Abstract

**Introduction:**

The Multiple primary malignant tumors (MPMT) refers to the occurrence of two primary malignant tumors in the same organ or organs in the same patient at the same time. However, MPMT is rare in the urinary system. Congenital urinary tract anomalies (e.g., duplex systems) predispose patients to recurrent infections, stones, and rare malignancies, creating diagnostic and therapeutic challenges.

**Case presentation:**

The patient was an elderly woman presenting with left low back pain and fever. Initial evaluation revealed left duplex kidney with ureteral malformation, complicated by upper kidney abscess, left kidney stone, and ureteropelvic junction stone. A ureteral stent was placed for symptom relief. After admission, enhanced CT/MRI identified left renal rupture with stone leakage, upper renal tumor, and ureteral tumor. Histopathology confirmed high-grade renal cell carcinoma (RCC) and urothelial carcinoma in situ (CIS), classified as a rare “duplication carcinoma.” Diagnostic delays occurred due to severe tumor necrosis masking imaging features, inconclusive initial endoscopy, and overlapping symptoms of systemic infection. The patient's complex anatomy (duplex kidney) and multifocal pathology (tumors, stones, infection) required multidisciplinary reevaluation to differentiate malignancy from inflammatory processes.

**Clinical discussion:**

This case is a high-grade renal cell carcinoma with urothelial carcinoma in situ, which meets the definition of repeat carcinoma. The pathogenesis of this case may be due to the development of malformations of the kidney and ureter combined with tumor compression of the collecting system, which will lead to long-term inadequate drainage and secondary stones, infection, etc. Early diagnosis is the key to improve the survival rate of patients with synchronous carcinomas. Enhanced CT combined with magnetic resonance imaging can be used for initial diagnosis.

**Conclusions:**

Synchronous carcinomas (synchronous RCC and urothelial CIS) in a duplex kidney with stones/pyonephrosis is exceptionally rare, emphasizing the need for vigilance in congenital anomalies. Complex cases demand tailored strategies, integrating urologic, oncologic, and radiologic expertise to address anatomical anomalies, infection, and malignancy simultaneously. Early suspicion of neoplasia in refractory cases is critical to avoid delays.

## Highlights

• The patient was an elderly woman with a chief complaint of left low back pain with fever. Initial evaluation revealed left duplex kidney with ureteral malformation, complicated by upper kidney abscess, left kidney stone, and ureteropelvic junction stone.
• Histopathology confirmed high-grade renal cell carcinoma (RCC) and urothelial carcinoma in situ (CIS), classified as a rare "duplication carcinoma." Diagnostic delays occurred due to severe tumor necrosis masking imaging features, inconclusive initial endoscopy, and overlapping symptoms of systemic infection.
• Duplication carcinoma (synchronous RCC and urothelial CIS) in a duplex kidney with stones/pyonephrosis is exceptionally rare, emphasizing the need for vigilance in congenital anomalies.

## 1 Introduction

Duplication of the renal pelvis and ureter is one of the malformations of the urinary system, with an incidence of about 0.8 % [1]. Duplex kidney ureter is a kind of congenital malformation of the kidney in which the affected kidney is composed of two parts, the upper and the lower half of the kidney, which are combined into a whole, with a common capsule, and a shallow groove on the surface separating the two, but the renal vessels, renal collecting system and ureter are separated separately. The male to female ratio is about 1: 2, the probability of bilateral simultaneous occurrence is about 20 % of all duplex kidney malformations, and the incidence of the left side is slightly higher than that of the right side [2]. Duplex kidney and ureter malformation were divided into complete and incomplete. Incomplete was three times more than complete, and the upper half of the kidney accounted for about one third of the ipsilateral renal function. Complete duplex kidney ureteral malformation refers to the normal and abnormal ureter opening in the bladder or other parts respectively, and the ureteral opening of the upper half kidney is generally located in the inner and lower part of the ureteral opening of the lower half kidney (Weigert-Meyer law) [3]. The types of renal tumors associated with duplex kidney malformation include renal cell carcinoma, malignant fibrous tissue tumor, renal sarcomatoid carcinoma, multilocular cystic renal cell carcinoma, and so on. Early diagnosis is very helpful for the treatment of the disease. Most patients are found in the imaging examination of the physical examination, and can be complicated by low back pain, recurrent urinary tract infection, hydronephrosis, calculi, etc. There are many cases of combined tumors, but the case of two primary malignant tumors is rare. The work has been reported in line with the SCARE criteria 2025 [4].

## 2 Case presentation

**Fig. 1:**
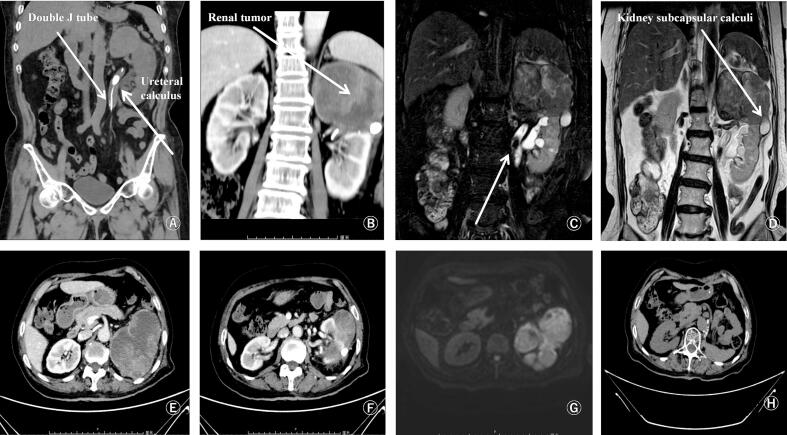
Preoperative and 6 month postoperative images. A: Preoperative CT scan showed left ureteral stent placement and left renal pelvis and ureter calculi. B: Preoperative enhanced CT showed rupture of the left upper kidney and renal subcapsular calculi. C, D, G: preoperative magnetic resonance imaging showed left duplex pelvis and ureter, upper ureteral calculi, renal subepithelial calculi, upper kidney and upper ureter occupying lesions; E, F: abnormal enhancement of the upper left kidney and ureter, possible mass; H: No abnormal CT scan was found 6 months after surgery.

The patient was a 78-year-old female with BMI of 17.31. The chief complaint was left low back pain with fever. One month ago, the patient underwent CT examination in a local hospital because of low back pain, which showed left pelvic ureteral malformation and stones at the ureteropelvic junction. There was no obvious blood supply in the upper half of the kidney on enhanced CT, and a left kidney abscess was considered. In order to relieve the patient's symptoms, a ureteral stent was placed under ureteroscopy in the local hospital. The symptoms of low back pain and fever were improved after treatment. The patient presented to our hospital for treatment of left renal calculi. After completing CT examination, left pyonephrosis was considered, and left ureteral J tube was placed before (Fig. 1A). In the upper left kidney, there was a localized cortical discontinuity and subcapsular fluid collection, with a ruptured kidney and a stone exposed (Fig. 1B). There was duplication of the pelvis and ureter on the left side, with abnormal enhancement of the upper left kidney and upper ureter, indicating a possible tumor (Fig. 1E, F). After antibiotic therapy, a follow-up urinary magnetic resonance examination revealed a reduction in the size of the left nephrotic lesion, an upper kidney and ureteral mass, renal rupture with exposed stones, and absorption of perirenal empyrosis (Fig. 1C, D, and G). Three urine cytology tests were negative, and cystoscopy showed no abnormality.

## 3 Treatment and outcome

**Fig. 2:**
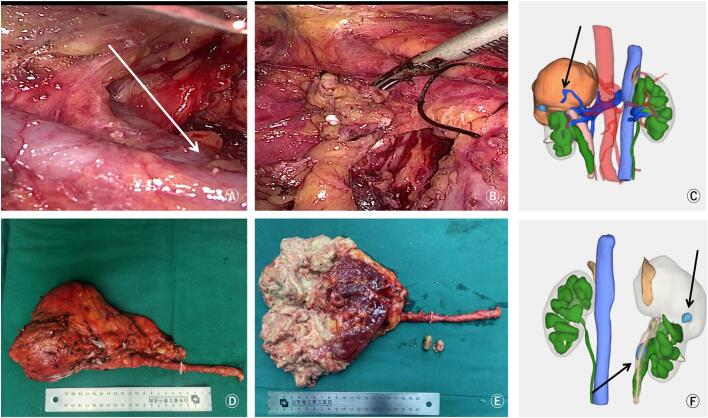
Intraoperative image and postoperative organ tissue specimen. A and B: The left incomplete duplication ureter showed a “Y” type bifurcation; C and F: preoperative 3D images showed duplex kidney and ureteral malformation, left kidney rupture with stone leakage, left upper kidney tumor and ureteral tumor; D, E: appearance of resected specimen and stone;

After full communication with family members of the patient and active preoperative preparation, resection of the unilateral repeated renal ureter was decided. The preoperative image 3D showed left duplication of renal pelvis and ureter malformation, left kidney duplication of upper kidney tumor, left kidney rupture with subcapsular calculus, and left ureteral calculus (Fig. 2C and F). Intraoperative exploration revealed incomplete duplication of the left ureter (Fig. 2B), complete resection of the left duplex kidney and ureter specimen (Fig. 2D), and longitudinal dissection of the renal pelvis showed that the renal tumor on the left duplex kidney showed to curd residue changes (Fig. 2E). After surgery, 8 cycles of epirubicin intravesical instillation chemotherapy were given to prevent bladder tumor implantation [5,6], and no abnormality was found in CT scan 6 months after surgery (Fig. 1H).

**Fig. 3:**
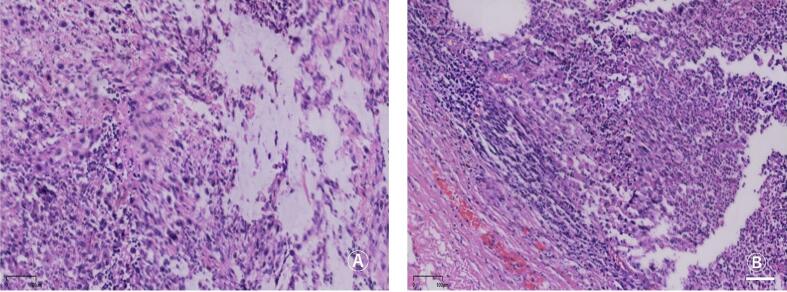
Postoperative pathological pictures. A: Kidney HE staining picture; B: Ureter HE staining picture.

Postoperative pathology showed renal high-grade renal cell carcinoma, NOS, nuclear grade 4 (WHO/ISUP) (Fig. 3A), with extensive necrosis. The cross section of the tumor was 11 ∗ 6.5 cm, invading the renal fibrous membrane and renal pelvis without involvement of perirenal fat capsule and renal sinus fat. Extensive geographic necrosis occupying 60–70 % of the tumor volume, predominantly localized to the central region. A distinct necrosis-viability transition zone was observed at the interface with residual clear cell renal cell carcinoma (CRCC; Fuhrman grade III) components. The periphery of necrotic foci exhibited infiltration by foamy macrophages and dystrophic calcification, indicative of chronic ischemic injury.

Ureteral tumor was found at the ureteropelvic junction, and the local ureteral mucosa was urothelial carcinoma in situ. There was focal ureteral wall fibrosis, hyalinization, and calcification (Fig. 3B). Focal mural fibrosis involving 30 % of the ureteral circumference at the ureteropelvic junction (UPJ) of the duplicated renal system, with associated CD68+ histiocytic infiltrates and α-SMA+ myofibroblast activation. These pathological features exhibit significant divergence from conventional RCC or urothelial carcinoma (UC): The spatial distribution and extent of necrosis exceed typical patterns in sporadic ccRCC, likely reflecting chronic hypoxia potentiated by obstructive malformation. The focal fibrotic response aligns mechanistically with obstructive uropathy rather than tumor-induced desmoplasia.

No carcinoma was found in the bladder, renal hilum, other tissues and adrenal gland, but in the lymph nodes around the renal hilum (0/7).

## 4 Discussion

Multiple primary malignant tumor (MPMT) refers to the occurrence of two primary malignant tumors in the same organ or organs in the same patient at the same time [[7], [8], [9]]. However, MPMT is rare in the urinary system. Warren proposed the diagnostic criteria of synchronous cancer in 1932 [9]: (1) each tumor must be proved to be malignant by histology; (2) Each has its own unique pathological morphology; (3) Metastatic cancer must be excluded; (4) The tumors occurred in different sites, and they were not consecutive. The pathogenesis of repeat cancer is still unclear, and may be related to genetics, immunodeficiency, radiotherapy and chemotherapy, unhealthy lifestyle, and mental factors [10,11]. Synchronous carcinoma represents a clinico-anatomic subtype of MPMT confined to duplicated renal and ureter systems. Shared microenvironmental drivers (obstructive uropathy/chronic inflammation) linking both malignancies (Warren & Gates criteria for MPMT).

Symeonidis et al. reported a case of the youngest patient ever recorded. An otherwise healthy 43-year-old military male with the chief complaint of left plank pain was seen in the office. Pathology confirmed the presence of a clear cell RCC and revealed an area of low-grade UC arising from the ipsilateral renal pelvis, not visible in the preoperative imaging [12]. For urologists and pathologists, when examining solid kidney masses, they must be vigilant about such cases. Additional treatment adjustments may be necessary, thereby changing the initial treatment plan. Ulamec et al. reported a case where a patient suffered from simultaneous renal cell carcinoma on the same side and urothelial carcinoma of the renal pelvis and ureter [13]. Currently, there is no evidence suggesting that the malignancy of these tumors is higher when they occur simultaneously or that they have a specific histological pattern. Compared to prior reports, this case demonstrates anatomically amplified carcinogenesis. This case is a high-grade renal cell carcinoma with urothelial carcinoma in situ, which meets the definition of synchronous carcinomas. The pathogenesis of this case may be due to the development of malformations of the kidney and ureter combined with tumor compression of the collecting system, which will lead to long-term inadequate drainage and secondary stones, infection, etc. Most space-occupying lesions of the urinary tract are urothelial carcinoma, and squamous cell carcinoma is rare [14]. Clinical studies have shown that long-term infection, calculi, and abuse of analgesics or chemotherapy drugs can induce the occurrence of squamous cell carcinoma in urothelial tissue [15,16].

Early diagnosis is the key to improve the survival rate of patients with synchronous carcinomas. Enhanced CT combined with magnetic resonance imaging can be used for initial diagnosis. CTU, IVP, MRU and other imaging in urothelial tumors also have high diagnostic value [17]. In this case, due to severe necrosis of the tumor lesion and atypical imaging examination, the diagnosis could not be confirmed by imaging examination. No obvious space-occupying lesions were found under ureteroscopy in the local hospital, and the patient had a serious systemic infection, which was consistent with the clinical manifestations of urinary stone infection, misleading the initial diagnosis of the whole disease and causing a certain delay in the whole treatment process.

## 5 Conclusion

The duplication of kidney and ureter malformation combined with renal tumor, ureteral tumor, renal calculi, renal rupture and infection is rare. The complex condition affects the doctor's initial diagnosis. For patients with duplication of kidney and ureteral malformation combined with renal tumor, nephron-sparing surgery can be considered when the tumor volume is small. For large tumors, depending on the location, upper or lower nephrectomy may be considered, along with resection of the corresponding part of the ureter. At 6 months after operation, the imaging examination showed no metastasis or recurrence, and cystoscopy showed no bladder tumor growth. In the case of upper urinary tract malformation complicated with multiple lesions such as duplicate carcinoma, individualized diagnosis and treatment are needed.

## Author contribution

MKS and BQX designed the research study and wrote the manuscript; YJS and LHL analyzed and interpreted the data; HZ and ZPY performed research. All authors reviewed the manuscript. All authors read and approved the final manuscript.

## Consent

Written informed consent was obtained from the patient for publication and any accompanying images. A copy of the written consent is available for review by the Editor-in-Chief of this journal on request.

## Ethical approval

The approval was approved by the Ethical Committee of Shandong Provincial Third Hospital (NO: SDSLSYLLSP20240215). The data of this study was published with the permission of the patient. The patient had been informed before undergoing surgery that this case would be used for the publication, and the patient had been informed that the surgical and pathological data would be used for the sharing of clinical experience. The characters with the patient's identity have been removed from the research results to ensure that personal privacy is not divulged and there is no risk to the patient.

## Guarantor

Yunji Sun accepted full responsibility for the work.

## Research registration number

Not applicable.

## Declaration of Generative AI and AI-assisted technologies in the writing process

The author(s) declare that no Generative AI was used in the creation of this manuscript.

## Funding

Not applicable.

## Conflict of interest statement

The authors declare that they have no competing interests.

## Data Availability

All data generated or analyzed during this study are included in this published article.
